# Prevalence of Vitamin D Deficiency and Associated Factors in Critically Ill Patients: A Multicenter Observational Study

**DOI:** 10.3389/fnut.2021.768804

**Published:** 2021-12-13

**Authors:** Kuo-Wei Chen, Chung-Wei Chen, Kuo-Ching Yuan, I-Ting Wang, Fang-Ming Hung, An-Yi Wang, Yin-Chin Wang, Yu-Ting Kuo, Yi-Che Lin, Ming-Chieh Shih, Yu-Chung Kung, Sheng-Yuan Ruan, Ching-Tang Chiu, Anne Chao, Yin-Yi Han, Li-Kuo Kuo, Yu-Chang Yeh

**Affiliations:** ^1^Department of Surgery, National Taiwan University Hospital Hsin-Chu Branch, Hsinchu, Taiwan; ^2^Department of Surgical Intensive Care Unit, Far Eastern Memorial Hospital, New Taipei City, Taiwan; ^3^Department of Critical Care Medicine, Taipei Medical University Hospital, Taipei, Taiwan; ^4^Division of Critical Care Medicine, Mackay Memorial Hospital, Taipei, Taiwan; ^5^Department of Emergency Medicine, School of Medicine, College of Medicine, Taipei Medical University Hospital, Taipei, Taiwan; ^6^Graduate Institute of Injury Prevention and Control, College of Public Health, Taipei Medical University, Taipei, Taiwan; ^7^Department of Anesthesiology, National Taiwan University Hospital, Taipei, Taiwan; ^8^Department of Environment and Occupational Medicine, National Taiwan University Hospital, Taipei, Taiwan; ^9^Department of Public Health, Institute of Epidemiology and Preventive Medicine, National Taiwan University, Taipei, Taiwan; ^10^Department of Medicine, National Taiwan University Hospital, Taipei, Taiwan; ^11^Department of Traumatology, National Taiwan University Hospital, Taipei, Taiwan; ^12^Department of Medicine, Mackay Medical College, New Taipei City, Taiwan

**Keywords:** vitamin D, critical care, deficiency, severity, mortality

## Abstract

**Background:** Vitamin D deficiency is common in the general population worldwide, and the prevalence and severity of vitamin D deficiency increase in critically ill patients. The prevalence of vitamin D deficiency in a community-based cohort in Northern Taiwan was 22.4%. This multicenter cohort study investigated the prevalence of vitamin D deficiency and associated factors in critically ill patients in Northern Taiwan.

**Methods:** Critically ill patients were enrolled and divided into five groups according to their length of stay at intensive care units (ICUs) during enrolment as follows: group 1, <2 days with expected short ICU stay; group 2, <2 days with expected long ICU stay; group 3, 3-7 days; group 4, 8-14 days; and group 5, 15-28 days. Vitamin D deficiency was defined as a serum 25-hydroxyvitamin D (25(OH)D) level < 20 ng/ml, and severe vitamin D deficiency was defined as a 25(OH)D level < 12 ng/ml. The primary analysis was the prevalence of vitamin D deficiency. The exploratory analyses were serial follow-up vitamin D levels in group 2, associated factors for vitamin D deficiency, and the effect of vitamin D deficiency on clinical outcomes in critically ill patients.

**Results:** The prevalence of vitamin D deficiency was 59% [95% confidence interval (CI) 55-62%], and the prevalence of severe vitamin D deficiency was 18% (95% CI 15-21%). The median vitamin D level for all enrolled critically ill patients was 18.3 (13.7-23.9) ng/ml. In group 2, the median vitamin D levels were <20 ng/ml during the serial follow-up. According to the multivariable analysis, young age, female gender, low albumin level, high parathyroid hormone (PTH) level, and high sequential organ failure assessment (SOFA) score were significantly associated risk factors for vitamin D deficiency. Patients with vitamin D deficiency had longer ventilator use duration and length of ICU stay. However, the 28- and 90-day mortality rate were not associated with vitamin D deficiency.

**Conclusions:** This study demonstrated that the prevalence of vitamin D deficiency is high in critically ill patients. Age, gender, albumin level, PTH level, and SOFA score were significantly associated with vitamin D deficiency in these patients.

## Introduction

Vitamin D is a pleiotropic hormone that regulates several crucial physiological functions (1, 2). Vitamin D deficiency is related to multiple health issues, including cardiovascular disease, cancer, metabolic syndrome and infection [1]. In the general population, the prevalence of vitamin D deficiency exists ranges from 20 to 80% (3–5), and in critical ill patients, the prevalence and severity of vitamin D deficiency increase. In addition to baseline vitamin D deficiency, decreased intake and absorption, increased losses and decreased production of vitamin D (6), enhanced conversion of 25-hydroxyvitamin D (25(OH)D) to the active 1,25-dihydroxyvitamin D3 (1,25(OH)2D) (7), and acute fluid shifts (8) may result in severely low vitamin D levels. Several observational studies have shown that vitamin D deficiency is associated with sepsis severity, organ dysfunction, and even mortality (9–15). However, numerous studies have reported that the effect of vitamin D deficiency on mortality is uncertain (16, 17).

In a community-based cohort in Northern Taiwan, the mean serum concentration of 25(OH)D was 28.9 ng/ml, and the prevalence of vitamin D deficiency (25(OH)D level < 20 ng/L) was 22.4% (18). Moreover, vitamin D deficiency might be related to area specific health issues and patients' characteristics (16, 19–22). Because of the worldwide concern of a high prevalence vitamin D deficiency and its effect on clinical outcomes in critically ill patients (23, 24), we conducted this multicenter observational study in Northern Taiwan to investigate the prevalence of vitamin D deficiency in critical ill patients and the associated factors of vitamin D deficiency. In addition, we perform a series of examinations to determine vitamin D levels in specific critically ill patients to follow up the change in their vitamin D levels in intensive care units (ICUs).

## Methods

### Study Design and Patient Enrolment

This multicenter, prospective, observational cohort study was approved by the Research Ethics Committee of National Taiwan University Hospital (approval number: 201805087RINB) and registered on the ClinicalTrials.gov protocol registration system (ID: NCT03639584). It was conducted at four hospitals in Northern Taiwan (north latitude 25:01– 25:03) between August 2018 and July 2020. Patients admitted to the ICUs were eligible for enrollment. Patients were excluded if they met the following exclusion criteria: aged <20 years, lower body mass index (< 18 kg/m^2^), severe anemia (hemoglobin level < 7 g/dl), received additional vitamin D supplement (>3,000 IU/day) within 4 weeks, previous admission to ICU within 3 months, hyperparathyroidism, rickets, liver cirrhosis (Child-Pugh C). Written informed consent was obtained from critically ill patients or patients' legally authorized representatives before enrolment in the study. In the four participating hospitals, the usual supplementing vitamin D was only from daily regular nutrition, mostly ranged from 800 to 2,000 IU. There was no routine high-dose vitamin D therapy in the 4 hospitals.

### Patient Grouping

For cross-sectional analysis of vitamin D deficiency, the enrolled critically ill patients were allocated to the following five groups according to their length of ICU stay at enrolment: group 1, <2 days and expected further ICU stay <5 days; group 2, <2 days and expected further ICU stay >5 days; group 3, ranging from 3 to 7 days; group 4, ranging from 8 to 14 days; and group 5, ranging from 15 to 28 days. Blood samples were obtained at enrolment, and serum 25(OH)D, parathyroid hormone (PTH), and cortisol levels were examined. In patients with sepsis, the procalcitonin level was examined. Subsequent time points (e.g., length of ICU-stay, 28-day mortality) were defined from the time on or after the day of enrolment.

For longitudinal follow-up of the vitamin D levels during the ICU stay since ICU admission, serial blood samples were obtained from patients in group 2 on day 7, 14, 21, and 28, and 25(OH)D levels were examined. If the patients died or were discharged from ICUs, further blood samples were not obtained. Patient characteristics, admission season, hemodynamic data, and laboratory data were recorded, and acute physiology and chronic health evaluation II (APACHE II) (25), Charlson et al. (26), and sequential organ failure assessment (SOFA) scores were calculated (27). The clinical outcomes were followed up for 90 days after patient enrolment, including duration of ventilator use, ICU-stay, and hospital stay, ICU-free days to day 28, and survival status.

### Examination and Category of Vitamin D Level

Blood serum samples were stored at −80°C. The serum 25(OH)D level was measured with the commercially available TOTAL Liaison chemiluminescence assay (Liaison, Diasorin S.p.A., Saluggia, Italy) (28). Accordingly, patients were classified into to the following four categories: sufficiency (≥30 ng/ml), insufficiency (<30 ng/ml), deficiency (<20 ng/ml), moderate deficiency (12-19.9 ng/ml), and severe deficiency (<12 ng/ml) (29).

### Primary Analysis and Exploratory Analyses

The primary analysis was the prevalence of vitamin D deficiency. The exploratory analyses included associated factors for vitamin D deficiency, serial vitamin D levels in group 2, and the effect of vitamin D deficiency on clinical outcomes of critically ill patients. Moreover, patients were divided into four groups according to their seasons at enrolment, and the median vitamin D levels were compared among the four seasons.

### Statistical Analysis

All statistical analyses were performed using SPSS version 20 (IBM, Armonk, NY, USA). Normally distributed numerical data were compared using one-way analysis of variance (ANOVA) and *post hoc* Tukey tests and are expressed as means (standard deviation). Non-normal distributed numerical data were compared using Kruskal-Wallis test with *post hoc* Mann-Whitney *U*-tests and are expressed as medians (interquartile range). Categorical variables were compared using chi-square tests or Fisher's exact tests as appropriate and are presented as percentages. Logistic regression models were used to estimate the relationship between the associated factors and vitamin D deficiency and the relationship between the vitamin D level and 28-day mortality rate. Backwards selection of variables was performed in the multivariable logistic regression. A *P-*value of <0.05 indicated a significant difference.

## Results

### Patient Enrolment and Characteristics

**Figure 1:**
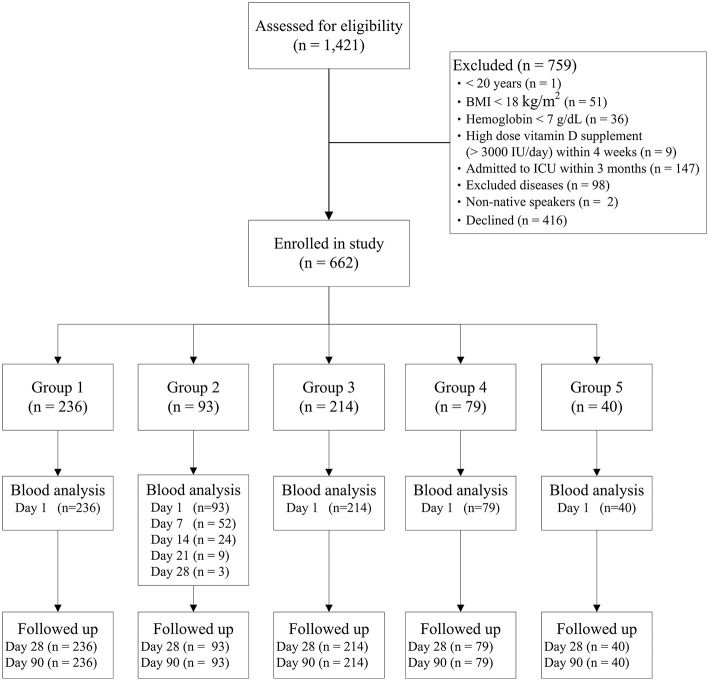
Flow chart of patient enrolment and grouping. BMI, body mass index; ICU, intensive care unit.

In total, 1,421 patients were assessed for enrolment eligibility, and 759 patients were excluded (Figure 1). Finally, 662 patients were enrolled and allocated to the five groups according to their length of ICU stay on the enrolment day, and all enrolled patients had completed their 90-day follow up. Characteristics of patients in the five groups are presented in Table 1. Of the patients enrolled, 50% were admitted to ICUs for postoperative care, and 18% had sepsis at enrolment. Furthermore, 55% of the enrolled patients were intubated and received mechanical ventilation at enrolment.

**Table 1:** Patient characteristics and clinical outcomes.

	**Total**	**Group 1**	**Group 2**	**Group 3**	**Group 4**	**Group 5**
Number	662	237	93	211	82	39
Age (years)	67 (56-78)	68 (55-78)	64 (54-74)	68 (58-79)	66 (57-80)	70 (59-78)
Gender (female/male)	257/405	106/131	29/64	76/135	32/50	14/25
Height (cm)	162 (155-169)	162 (155-168)	165 (158-171)	163 (156-168)	164 (155-170)	161 (155-168)
Weight (kg)	65 (56.1-74.8)	64.7 (55-73.1)	65 (56.8-77.8)	65.6 (57.5-74.5)	64.5 (55.4-77.1)	64 (55.3-79)
BMI (kg/m^2^)	24 (21.5-27.2)	23.8 (21.6-27.2)	24.1 (21-28.4)	24.2 (21.7-26.6)	23.8 (21.5-27.4)	24.2 (22.5-28.9)
APACHE II	15 (11-19)	14 (10-19)	21 (15-26)	20 (16-25)	19 (15-24)	20 (15-24)
SOFA score	5 (3-7)	3 (2-5)	7 (4-10)	5 (3-8)	5 (3-8)	5 (4-9)
Charlson score	2 (1-4)	2 (1-4)	2 (0-4)	3 (1-4)	2 (0-4)	2 (1-5)
ICU admission						
Sepsis	117 (18%)	16 (7%)	26 (28%)	39 (19%)	24 (29%)	12 (31%)
Postoperative	330 (50%)	150 (63%)	34 (37%)	98 (46%)	35 (43%)	13 (33%)
Respiratory	54 (8%)	14 (6%)	10 (11%)	23 (11%)	3 (4%)	4 (10%)
Cardiovascular	33 (5%)	16 (7%)	1 (1%)	11 (5%)	3 (4%)	2 (5%)
Neurologic	63 (10%)	15 (6%)	10 (11%)	25 (12%)	10 (12%)	3 (8%)
Others	65 (10%)	26 (11%)	12 (13%)	15 (7%)	7 (9%)	5 (13%)
Comorbidities						
Hypertension	90 (14%)	24 (10%)	18 (19%)	28 (13%)	16 (20%)	4 (10%)
Diabetes	202 (31%)	64 (27%)	25 (27%)	78 (37%)	21 (26%)	14 (36%)
Liver diseases	108 (16%)	40 (17%)	14 (15%)	40 (19%)	11 (13%)	3 (8%)
Renal diseases	100 (15%)	26 (11%)	17 (18%)	31 (15%)	17 (21%)	9 (23%)
Hemodialysis	35 (5%)	11 (5%)	6 (7%)	10 (5%)	5 (6%)	3 (8%)
Metastasis	48 (7%)	24 (10%)	4 (4%)	15 (7%)	2 (2%)	3 (8%)
PTH (pg/ml)	30 (19-49)	29 (19-42)	37 (24-65)	30 (18-48)	29 (18-59)	28 (17-44)
Vitamin D (ng/ml)	18.3 (13.7-23.9)	19.9 (14.8-26.1)	18.1 (13-23.1)	17.5 (12.4-22.7)	17.5 (14.2-24.9)	18.1(13.5-22.9)
>30	66 (10%)	35 (15%)	6 (7%)	18 (8%)	5 (6%)	2 (5%)
20.1-30	208 (31%)	80 (34%)	32 (34%)	58 (28%)	26 (32%)	12 (31%)
12.1-20	268 (41%)	92 (39%)	35 (38%)	88 (42%)	35 (43%)	18 (46%)
≤ 12	120 (18%)	30 (12%)	20 (21%)	47 (22%)	16 (19%)	7 (18%)
After enrolment						
ICU stay (days)	8 (4-8)	3 (2-4)	9 (6-18)	10 (6-19)	20 (14-26)	28 (21-39)
Hospital stay (days)	28 (17-47)	18 (11-29)	30 (17-48)	34 (21-52)	43 (27-60)	53 (36-75)
28-day ICU free days	25 (18-27)	26 (25-27)	21 (15-25)	24 (16-27)	23 (14-26)	18 (5-26)
28-day mortality	59 (9%)	9 (4%)	14 (15%)	21 (10%)	12 (15%)	3 (8%)
90-day mortality	105 (16%)	17 (7%)	21 (23%)	38 (18%)	21 (26%)	10 (26%)

*Data are presented as number, number (%), or median (interquartile range)*.

*APACHE, acute physiology and chronic health evaluation; BMI, body mass index; ICU, intensive care unit; PTH, parathyroid hormone; SOFA, sequential organ failure assessment*.

### Prevalence and Characteristics of Patients With Vitamin D Deficiency

The prevalence of vitamin D deficiency and clinical outcomes of the five groups are shown in Table 1. The overall prevalence of vitamin D deficiency was 59% [95% confidence interval (CI) 55-62%], and the prevalence of severe vitamin D deficiency was 18% (95% CI 15-21%). The median vitamin D level for all enrolled critically ill patients was 18.3 (13.7-23.9) ng/ml. The proportion of sepsis is higher in the patients with severe deficiency vitamin D than in those with sufficiency of vitamin D (30 vs. 15%, *P* = 0.032). The median vitamin D levels differed significantly among patients enrolled during different seasons (Spring 17.6 [13-23.6]; Summer 17.5 [12.4-22.4]; Fall 19.2 [14.8-23.6]; and Winter 19.1 [14.8-27] ng/ml; *P* = 0.028). More patients with sepsis were admitted in Spring than in Fall (25 vs. 11%, *P* = 0.001), and more patients with neurologic diseases were admitted in Winter than in Summer (17 vs. 6%, *P* < 0.001).

Moreover, patients were allocated to four categories according to their vitamin D levels at enrolment, and patient characteristics and clinical outcomes are shown in Table 2. The procalcitonin levels in patients with sepsis did not differ significantly between the four groups (sufficiency, 3.9 [0.5–60.6]; insufficiency, 4.5 [0.4-26.1]; moderate deficiency, 4.7 [0.6-14.6]; and severe deficiency, 2.1 [0.5-11.2] ng/ml; *P* = 0.722). The 28- and 90-day mortality did not differ significantly among the four categories. Patients with vitamin D deficiency were associated with longer duration of ventilator use and longer length of ICU stay. Furthermore, ICU-free days to day 28 were more in the vitamin D sufficiency group than in the vitamin D insufficiency, moderate deficiency, and severe deficiency groups. Length of hospital stay did not differ significantly among the four categories.

**Table 2:** Characteristics and clinical outcomes of patients in different categories of vitamin D level.

**Vitamin D level**	**Sufficiency**	**Insufficiency**	**Moderate deficiency**	**Severe Deficiency**	
**(ng/ml)**	**>30**	**20.1-30**	**12.1-20**	**≤12**	***P***
Number	66	208	268	120	
Age (years)	73 (65-83)	70 (60-79)^*^	67 (55-78)^*^	61 (50-72)^*^	<0.001
≤ 56	9 (14%)	39 (19%)	73 (27%)	50 (42%)	<0.001
>56, ≤ 67	10 (15%)	44 (21%)	60 (22%)	32 (27%)	0.330
>67, ≤ 78	19 (29%)	64 (31%)	64 (24%)	19 (16%)	0.021
>78	28 (42%)	61 (29%)	71 (27%)	19 (16%)	0.001
BMI (kg/m^2^)	24.3 (3.6)	24.5 (4.6)	25.2 (5.4)	24.8 (5.3)	0.458
ICU admission					
Sepsis	10 (15%)	30 (14%)	41 (15%)	36 (30%)	0.002
Postoperative	37 (56%)	113 (54%)	137 (51%)	43 (36%)	0.006
Respiratory	8 (12%)	13 (6%)	23 (9%)	10 (8%)	0.482
Cardiovascular	4 (6%)	9 (4%)	13 (5%)	7 (6%)	0.909
Neurologic	7 (11%)	25 (12%)	22 (8%)	9 (8%)	0.440
Others	4 (6%)	22 (11%)	38 (14%)	18 (15%)	0.203
Seasons					
Spring	21 (32%)	45 (22%)	68 (25%)	39 (33%)	0.117
Summer	11 (17%)	46 (22%)	70 (26%)	35 (29%)	0.201
Fall	10 (15%)	71 (34%)	72 (27%)	22 (18%)	0.002
Winter	22 (36%)	46 (22%)	58 (22%)	24 (20%)	0.054
Hospitals					
Site 1	38 (58%)	113 (54%)	132 (49%)	44 (37%)	0.009
Site 2	15 (23%)	50 (24%)	64 (24%)	43 (36%)	0.069
Site 3	9 (14%)	25 (12%)	48 (18%)	18 (15%)	0.359
Site 4	4 (6%)	20 (10%)	24 (9%)	15 (13%)	0.546
APACHE II	15 (10-19)	15 (10-19)	15 (11-20)	16 (11-20)	0.419
SOFA score	5 (2-6)	4 (2-7)	5 (3-8)	5 (3-8)	0.127
MAP (mm Hg)	84 (76-92)	87 (78-96)^#^	88 (78-98)^#^	92 (82-99)^*^	0.016
Heart rate (breaths/min)	87 (75-101)	86 (74-100)^#^	86 (75-100)^#^	92 (82-102)	0.045
Creatinine (mg/dl)	1.1 (0.8-1.8)	0.9 (0.7-1.4)	1 (0.6-1.7)	1 (0.6-2.4)	0.131
White blood cells (k/μl)	11.3 (7.9-14.2)	10.6 (8.3-14.3)	10.7 (8-15)	10.4 (7.9-13.5)	0.922
Hematocrit (%)	30.8 (28-34)	30.8 (27.4-35.5)	30.1 (27.4-34.4)	29.6 (26.4-33.6)	0.125
Platelet (k/μl)	202 (141-273)	181 (130-253)	178 (123-253)	162 (110-227)	0.075
Lactate (mmol/L)	1.6 (1-2.5)	1.8 (1.2-6.6)	2 (1.3-7.3)^*^	2.2 (1.3-11.6)	0.045
Total bilirubin (mg/dl)	0.8 (0.5-1.2)	0.9 (0.6-1.3)	0.8 (0.5-1.4)	0.7 (0.5-1.8)	0.940
Albumin (g/dl)	3.1 (2.8-3.5)	3.1 (2.7-3.6)^#^	3.1 (2.7-3.5)^#^	2.9 (2.4-3.3)^*^	0.007
C-reactive protein (mg/L)	3.8 (2.4-20.1)	6.2 (1.6-15.8)	7.3 (2.6-16.9)	8.9 (3.4-17.1)	0.308
PTH (pg/ml)	26 (17-42)	25 (17-40)	31 (21-55)^*^	35 (25-65)^*^	<0.001
Cortisol (μg/dl)	21 (13-28)	19 (12-27)	18 (12-27)	18 (13-29)	0.877
Total Calcium (mmol/L)	2.1 (2-2.2)	2.1 (2-2.2)	2.1 (2-2.2)	2.1 (2-2.2)	0.021
Duration of ventilator use (day)	2 (1-9)	5 (1-15)	8 (2-17)^*^	9 (3-26)^*^	0.001
ICU stay (days)	5 (3-8)	9 (3-19)^*^	9 (4-18)^*^	9 (4-19)^*^	0.001
28-day ICU free days	26 (24-27)	24 (18-27)^*^	25 (18-27)^*^	24 (18-27)^*^	0.011
Hospital stay (days)	27 (15-37)	26 (17-47)	28 (16-50)	32 (17-49)	0.444
28-day mortality	5 (8%)	17 (8%)	28 (10%)	9 (8%)	0.714
90-day mortality	10 (15%)	32 (15%)	42 (16%)	23 (19%)	0.727

*Data are presented as number, number (%), mean (standard deviation), or median (interquartile range). P values are for one-way analysis of variance among the four categories. Duration of ventilator use in intubated patients are compared among the four categories*.

* *compared with group sufficiency (>30) and*

# *compared with group severe insufficiency (≤12) represent a significant difference between the two groups using post hoc analysis with Tukey test or Mann–Whitney U-test*.

*APACHE, acute physiology and chronic health evaluation; BMI, body mass index; MAP, mean arterial pressure; ICU, intensive care unit; PTH, parathyroid hormone; SOFA, sequential organ failure assessment*.

### Serial Follow-Up of Vitamin D Levels

**Figure 2:**
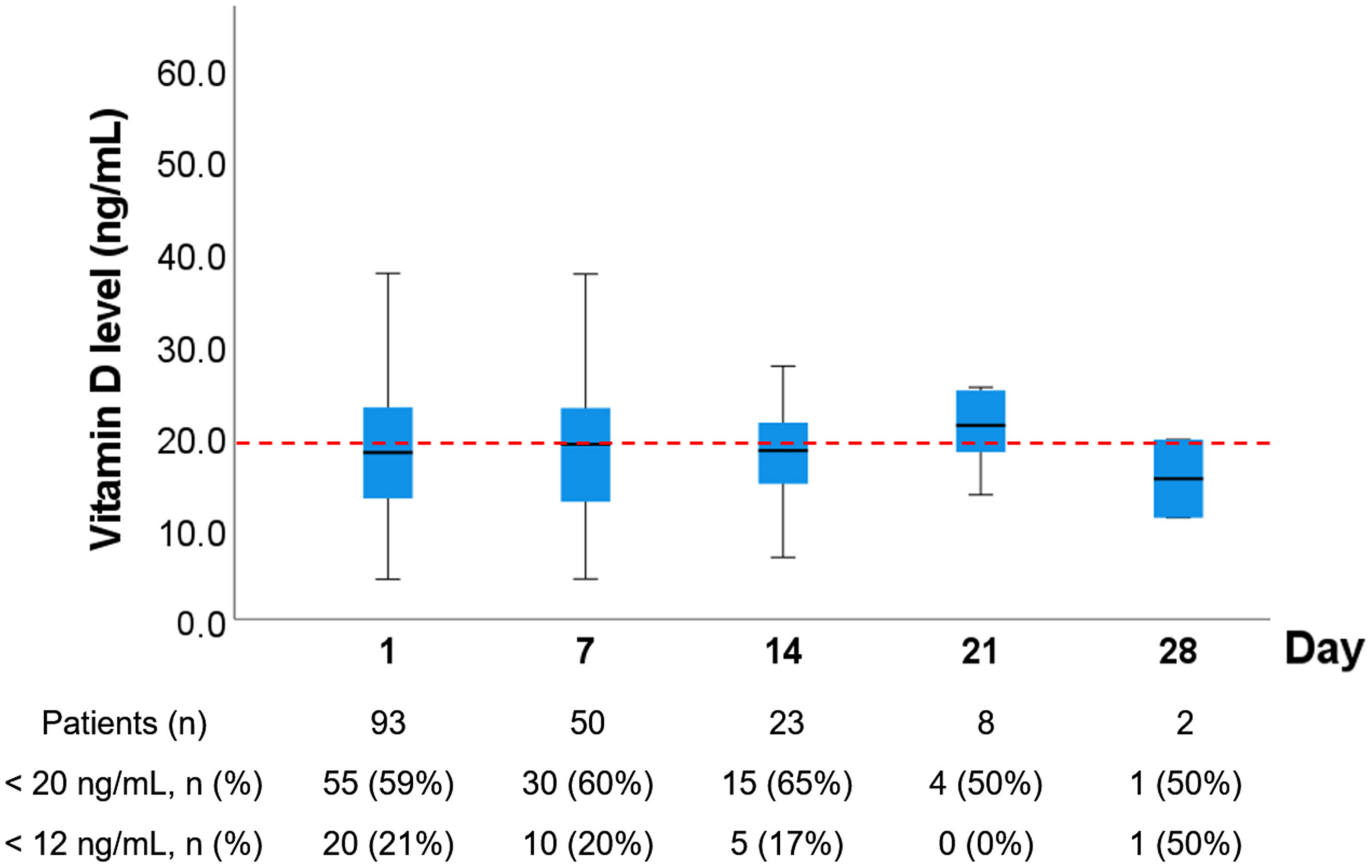
Serial follow-up of vitamin D levels in Group 2. Serial blood samples were obtained from patients in group 2 on day 7, 14, 21, and 28. If the patients died or were discharged from ICUs, further blood samples were not obtained. If the patients received high-dose vitamin D therapy, the subsequent vitamin D level of these patients were excluded One patient received high-dose vitamin D (560,000 IU) therapy within Day 7, and the vitamin D levels of this patient on day 1 and 7 was 12.4 and 78.6 ng/ml, respectively. This patient was discharger before day 14. Another patient received high-dose vitamin D (560,000 IU) therapy between Day 7 and 14, and the vitamin D levels of this patient on day 1, 7, 14, 21, and 28 were 13.1, 18.8, 92, 68.2, and 64.6 ng/ml, respectively.

The vitamin D levels at day 1, 7, 14, 21, and 28 of group 2 are shown in Figure 2. The prevalence of vitamin D deficiency was 59% on day 1, 60% on day 7, 55% on day 14, 50% on day 21, and 100% on day 28. Five patients in group 2 receive high-dose vitamin D supplement. Three patients were discharged before day 7. One patient received high-dose vitamin D (560,000 IU) therapy within Day 7, and the vitamin D levels of this patient on day 1 and 7 was 12.4 and 78.6 ng/ml, respectively. This patient was discharger before day 14. One patient received high-dose vitamin D between day 7 and 14 and had a complete serial follow-up of vitamin D levels as follows: day 1, 13.1 ng/ml; day 7, 18.8 ng/ml; day 14, 92 ng/ml; day 21, 68.2 ng/ml, and day 28, 64.6 ng/ml.

### Associated Factors for Vitamin D Deficiency and Mortality

The associated factors for vitamin D deficiency based on univariable and multivariable analyses are presented in Table 3. In the multivariable analysis, young age, female gender, low albumin level, high PTH level, and high SOFA scores were significantly associated risk factors for vitamin D deficiency. The associated factors for 28-day mortality based on univariable and multivariable analyses are presented in Table 4. Both univariable and multivariable analyses revealed no association between the vitamin D level and 28-day mortality. In the multivariable analysis, age, high heart rate, and high SOFA score were significantly associated risk factors for 28-day mortality.

**Table 3:** Associated factors for vitamin D deficiency (<20 ng/ml).

	**Univariable**			**Multivariable**		
	**OR**	**95% CI**	***P***	**OR**	**95% CI**	***P***
Age (years)	0.979	0.969-0.989	<0.001	0.969	0.958-0.980	<0.001
Female	1.676	1.212-2.317	0.002	2.098	1.479-2.976	<0.001
Albumin (g/dl)	0.692	0.530-0.903	0.007	0.646	0.487-0.857	0.002
PTH (pg/ml)	1.005	1.002-1.009	0.003	1.006	1.002-1.010	0.002
SOFA score	1.059	1.012-1.107	0.012	1.066	1.014-1.120	0.012
APACHE II score	1.022	0.996-1.048	0.104			
BMI (kg/m^2^)	1.024	0.992-1.057	0.182			
Heart rate (bpm)	1.009	0.999-1.018	0.071			
MAP (mmHg)	1.009	0.998-1.020	0.118			
Platelet (k/μl)	0.999	0.997-1.000	0.152			
Creatinine (mg/dl)	1.080	0.993-1.174	0.072			
Sepsis	1.448	0.954-2.200	0.082			
Post-operation	0.715	0.524-0.976	0.034			

*Backwards selection of variables was performed in the multivariable logistic regression. APACHE, acute physiology and chronic health evaluation; BMI, body mass index; CI, confidence interval; MAP, mean arterial pressure; OR, odds ratio; PTH, Parathyroid Hormone; SOFA, sequential organ failure assessment*.

**Table 4:** Associated factors for 28-day mortality.

	**Univariable**			**Multivariable**		
	**OR**	**95% CI**	***P***	**OR**	**95% CI**	***P***
Age (years)	1.039	1.019-1.060	<0.001	1.043	1.020-1.066	<0.001
Heart rate (bpm)	1.039	1.022-1.056	<0.001	1.052	1.033-1.071	<0.001
SOFA score	1.274	1.189-1.364	<0.001	1.302	1.205-1.405	<0.001
Albumin (g/dl)	0.493	0.316-0.769	0.002			
Vitamin D (ng/ml)	0.990	0.959-1.023	0.551			
PTH (pg/mL)	1.006	1.002-1.009	0.001			
APACHE II score	1.051	1.008-1.097	0.020			
Charlson score	1.140	1.037-1.253	0.006			
Total bilirubin (mg/dl)	1.133	1.057-1.214	<0.001			
Lactate (mmol/L)	1.059	1.090-1.089	<0.001			
C-reactive protein (mg/L)	1.038	1.010-1.068	0.009			
Cortisol (μg/dl)	1.018	1.004-1.032	0.013			
Creatinine (mg/dl)	1.065	0.977-1.160	0.151			
MAP (mmHg)	0.975	0.956-0.995	0.015			
Hematocrit (%)	0.949	0.900-1.000	0.049			
Platelet (k/μl)	0.995	0.992-0.999	0.004			

*Backwards selection of variables was performed in the multivariable logistic regression. APACHE, acute physiology and chronic health evaluation; CI, confidence interval; MAP, mean arterial pressure; OR, odds ratio; PTH, parathyroid Hormone; SOFA, sequential organ failure assessment*.

## Discussion

This study shows that the prevalence of vitamin D deficiency is high (59%) in critically ill patients, and only 10% of critically ill patients had a vitamin D level > 30 ng/ml. Furthermore, we found that vitamin D deficiency is associated with long durations of ventilator use and ICU stay, but is not associated with 28- or 90-day mortality. Moreover, this study revealed that young age, female gender, low albumin level, high PTH level, and high SOFA score are associated with vitamin D deficiency. In the serial examinations of vitamin D level, we further found that the median vitamin D level remained <20 ng/dl in most critically ill patients during their stay in ICUs.

The median vitamin D level (18.3 ng/ml) in the critically ill patients of this study was <28.9 ng/ml in the participants of a community-based cohort study in Northern Taiwan (18). The prevalence of vitamin D deficiency was higher in critically ill patients than in community participants. One associated factor of vitamin D deficiency of this study was a high PTH level indicates that some critically ill patients had chronic vitamin D deficiency before ICU admission (30–32). These two studies shared two associated factors of vitamin D deficiency, namely young age and female gender. Younger and female adults might use more sun protection and have more indoor activities than elderly patients (33, 34). A similar finding in an urban Korean hospital showed that vitamin D levels were higher in older critically ill surgical patients (35). Moreover, the type and severity of critical illness between young and elderly patients may be different, and further studies are warranted to investigate the differences and figure out the complicated effects of age and sex on Vitamin D deficiency in critically ill patients. Two other associated factors for vitamin D deficiency in this study were low albumin level (hypoalbuminemia) and high SOFA score. Hypoalbuminemia as an associated factor for vitamin D deficiency was revealed in a previous study (14), and their multivariable analysis revealed that serum albumin level was associated with vitamin D deficiency with an OR of 0.92 (95% CI 0.87-0.97). We suggest that albumin and vitamin D deficiency are associated with several clinical conditions, including chronic intake insufficiency and acute consumption. SOFA score has been reported to be associated with vitamin D deficiency in several previous studies (14, 32), and it may represent acute consumption or decreased production of vitamin D in patients with a high SOFA score.

In this study, vitamin D deficiency was associated with a longer duration of ventilator use and longer length of ICU stay but was not associated with the length of hospital stay or mortality. To avoid the influence of non-survived critically ill patients with short ICU stay, we also compared the ICU-free days to day 28 among patients with different vitamin D levels and confirmed that vitamin D deficiency was associated with less ICU-free days. This finding is compatible with a previous study (32). Although a systemic review and meta-analysis suggested that vitamin D deficiency increases the mortality risk of critically ill patients (11), our study and other recent studies have reported an uncertain relationship with mortality (16). We suggest that vitamin D deficiency is affected by multiple factors and may have different contributions to mortality in different diseases or in different populations of critically ill patients. Further studies are warranted to investigate the effect of vitamin D deficiency on clinical outcomes in different populations of critically ill patients.

This study has several limitations. First, the number of patients in this study was not sufficient to analyze the effect of vitamin D deficiency on clinical outcomes in the subgroups of specific diseases or populations. Second, this observational study did not limit the use of higher daily supplement of vitamin D (>2,000 IU/day) or high-dose vitamin D3 (>540,000 IU) in these patients during their ICU stay (29, 36). In total, 10 patients received high-dose vitamin D3 during their ICU stay. Third, information regarding the outdoor activity of patients and the ICU environment of sunlight exposure was not included and analyzed in this study. Forth, the effects of age, obesity, and seasons on the vitamin D levels might be confounded by different diseases or diagnoses at ICU admission, and careful subgroup analysis with more patients is recommended in further investigations. This study has two strengths. First, this was a multicenter study that included different areas of Northern Taiwan. Second, a serial follow-up of vitamin D levels was performed for group 2, and it indicated that normal daily supplement of vitamin D were not sufficient to increase the vitamin D level to >30 ng/ml. Further studies are warranted to investigate the effects of daily supplement or high-dose vitamin D on serum vitamin D level and clinical outcomes in critically ill patients.

## Conclusions

Our results demonstrated that the prevalence of vitamin D deficiency is high in critically ill patients in Northern Taiwan. Young age, female gender, low albumin level, high PTH level, and high SOFA score are significantly associated risk factors for vitamin D deficiency. Further studies in different countries or areas are warranted and crucial for investigating the prevalence, associated factors, and clinical impacts of vitamin D deficiency in different regions.

## Data Availability Statement

The raw data supporting the conclusions of this article will be made available by the authors upon reasonable request.

## Ethics Statement

The studies involving human participants were reviewed and approved by Research Ethics Committee of National Taiwan University Hospital. The patients/participants provided their written informed consent to participate in this study.

## Author Contributions

K-WC, C-WC, K-CY, L-KK, and Y-CY: concept and design. C-WC, K-CY, I-TW, F-MH, A-YW, Y-CW, Y-CK, S-YR, C-TC, AC, Y-YH, L-KK, and Y-CY: patient enrollment and data collection. K-WC, Y-TK, Y-CL, and Y-CY: statistics and data interpretation. K-WC, L-KK, and Y-CY: drafting manuscript. L-KK and Y-CY: study supervision. All authors: critical revision of the manuscript.

## Funding

This work was supported, in part, by grant from the National Taiwan University Hospital (108-S4130).

## Conflict of Interest

The authors declare that the research was conducted in the absence of any commercial or financial relationships that could be construed as a potential conflict of interest.

## Publisher's Note

All claims expressed in this article are solely those of the authors and do not necessarily represent those of their affiliated organizations, or those of the publisher, the editors and the reviewers. Any product that may be evaluated in this article, or claim that may be made by its manufacturer, is not guaranteed or endorsed by the publisher.
